# Genetic affinities among the historical provinces of Romania and Central Europe as revealed by an mtDNA analysis

**DOI:** 10.1186/s12863-017-0487-5

**Published:** 2017-03-07

**Authors:** Relu Cocoş, Sorina Schipor, Montserrat Hervella, Petru Cianga, Roxana Popescu, Claudia Bănescu, Mihai Constantinescu, Alina Martinescu, Florina Raicu

**Affiliations:** 1 “Carol Davila” University of Medicine and Pharmacy, Chair of Medical Genetics, 19-21, Prof. dr. Dimitrie Gerota St., 020032 Bucharest, Romania; 2 “Francisc I. Rainer” Institute of Anthropology, Romanian Academy, Bucharest, Romania; 3 Genome Life Research Center, Bucharest, Romania; 4 National Institute of Endocrinology “C. I. Parhon”, Bucharest, Romania; 5 Department of Genetics, Physical Anthropology and Animal Physiology, University of the Basque Country (UPV/EHU), Bizkaia, Spain; 6 Department of Immunology, Grigore T. Popa University of Medicine and Pharmacy, Iasi, Romania; 7 “Victor Babeş” University of Medicine and Pharmacy, Timişoara, Romania; 8 Department of Medical Genetics, University of Medicine and Pharmacy Tîrgu Mureş, Tîrgu Mureş, Romania; 9 Department of Medical Genetics, Ovidius University, Faculty of Medicine, Constanța, Romania

**Keywords:** Mitochondrial DNA, Romanian provinces, Genetic diversity, Transylvania

## Abstract

**Background:**

As a major crossroads between Asia and Europe, Romania has experienced continuous migration and invasion episodes. The precise routes may have been shaped by the topology of the territory and had diverse impacts on the genetic structure of mitochondrial DNA (mtDNA) in historical Romanian provinces. We studied 714 Romanians from all historical provinces, Wallachia, Dobrudja, Moldavia, and Transylvania, by analyzing the mtDNA control region and coding markers to encompass the complete landscape of mtDNA haplogroups.

**Results:**

We observed a homogenous distribution of the majority of haplogroups among the Romanian provinces and a clear association with the European populations. A principal component analysis and multidimensional scaling analysis supported the genetic similarity of the Wallachia, Moldavia, and Dobrudja groups with the Balkans, while the Transylvania population was closely related to Central European groups. These findings could be explained by the topology of the Romanian territory, where the Carpathian Arch played an important role in migration patterns. Signals of Asian maternal lineages were observed in all Romanian historical provinces, indicating gene flow along the migration routes through East Asia and Europe.

**Conclusions:**

Our current findings based on the mtDNA analysis of populations in historical provinces of Romania suggest similarity between populations in Transylvania and Central Europe, supported both by the observed clines in haplogroup frequencies for several European and Asian maternal lineages and MDS analyses.

**Electronic supplementary material:**

The online version of this article (doi:10.1186/s12863-017-0487-5) contains supplementary material, which is available to authorized users.

## Background

Romania is located in Southeastern Europe as part of the Central basin of the Black Sea and lower Danube basin, and the country is divided in the center by the orographic Carpathian-Balkan system. As a segment of the Danube basin, this territory was a major crossroads between Asia and Southeastern, Central, and North Europe and one of the direct eastward routes linking to the North Pontic steppe. The Romanian language is a part of the Eastern Romance languages that evolved from spoken Latin during the process of Romanization followed by a period of Slavic influence at the beginning of the sixth century [1].

The earliest anatomically modern human fossils in Europe attributed to the end of the Upper Paleolithic period were found in Romania in the Peştera cu Oase (the cave with bones) dated ~35 ka ^14^C BP [2] and Peștera Muierii (~30 ka ^14^C BP) [3].

Population movement during the Neolithic and Bronze Age shaped genetic variation in the present-day Romanian population, especially during the Middle Neolithic [4]. During the Iron Age, in the territory defined by the Danube Basin and Carpathian Arch, archaeological records and historical sources have revealed the presence of Indo-European populations close to Thracians who probably arrived during the second millennium BC named Dacians in Transylvania and Getae in Wallachia, Dobrudja, and Bessarabia [5].

The partial conquest of Dacia by Romans (1^st^ to 2^nd^ centuries AD), with the exception of current regions of Moldavia, northeastern Transylvania, and eastern Wallachia, was followed by a period of colonization by various groups from the Roman Empire (Italians, Illyrians, Thracians, Greeks, Celts, Germans, and Eastern or North Africans) who settled mostly in Transylvania [6].

Starting in the 6^th^ century, the Slavs extensively penetrated the present-day territory of Romania and were eventually assimilated into Daco-Romanian, proto-Romanian, and Romanian populations by the end of the 12^th^ century [6].

The current territory of Romania was divided in the Middle Ages (ca. 14^th^ century AD) into three different political structures: Wallachia, Moldavia, and Transylvania. Dobrudja was, during most of its medieval history, under Ottoman rule. During the next several centuries, the historical provinces of Romania were continuously under the suzerainty of the Byzantine, Austro-Hungarian, Habsburg, Ottoman, and Russian Empires. However, these empires had no major demographic impact, except in Transylvania, which underwent a massive colonization by Székelys, Saxons, and Hungarians under the domination of the Kingdom of Hungary between the 10^th^ and 13^th^ centuries [7].

The historical provinces evolved more or less separately for over 500 years under different political and religious influences, until the unification into a single country officially named Romania in the 19^th^ century.

At present, few studies have assessed the genetic composition of Romanian populations based on mitochondrial DNA (mtDNA) haplogroups. MtDNA variation in Romanian populations has mainly been analyzed in multinational studies characterized by a relatively small number of samples collected over the entire Romanian territory to encompass the complete mtDNA haplogroup landscape. Previous studies have revealed that Romanian populations exhibit genetic similarity with other Europeans [8–11], while another study pointed to possible segregation within the Middle East populations [12]. MtDNA variation in Romania was examined in a recent study, but the sample size was small, especially for the Dobrudja and Transylvania regions, and a detailed comparison of historical regions was not performed [13]. Other studies have focused on the mtDNA diversity of various minority groups, such as Aromanian, Hungarian, and Roma populations [10, 14–16].

The maternal lineages of Balkan Peninsula populations exhibit genetic uniformity, with region-specific characteristics [10]. It is worth noting that the resemblance between Western Balkan populations follows a nearest neighbor-based model that is in concordance with the geographical distribution [17].

To address the scarcity of information and to improve our understanding of mtDNA variation in the context of European and Middle East populations, we analyzed 714 Romanians belonging to the four historical provinces. We purpose to reveal the specific genetic differences between populations from the historical provinces that could experience demographic combinations as a consequences of past migrations, local admixture and colonization events in their past.

## Methods

### Ethics

Written informed consent was obtained for the collection of samples and subsequent analyses before patients were entered in the study, in accordance with protocols that comply with the Declaration of Helsinki, and the study was approved by the ethics committee of the “Carol Davila” University of Medicine and Pharmacy in Bucharest, Romania.

### Population data set

The samples analyzed in this study originated from the main historical provinces, Wallachia (*n* = 226), Dobrudja (*n* = 46), Moldavia (*n* = 235), and Transylvania (*n* = 207). The samples were randomly collected from maternally unrelated subjects with known genealogical information for at least three generations (parents and grandparents). For all samples, the precise affiliations to the Romanian counties (*n* = 41) that constitute the administrative divisions of present-day Romania were known (Additional file 1: Table S1).

### Mitochondrial DNA sequencing and haplotype identification

Genomic DNA was extracted from 200 μL of whole blood in EDTA or buccal swabs using the PureLink Genomic DNA Mini Kit (Invitrogen, Carlsbad, CA, USA). For all samples, the hypervariable segments HVS I (positions 16024–16416) and HVS II (positions 1–410) of the mtDNA control region were sequenced using two pairs of primers, as previously described [11].

PCRs were performed in 25 μL of reaction mixture including 1× PCR buffer (15 mM Tris–HCl, pH 8.0, and 50 mM KCl), 200 μM each dNTP, 2.5 mM MgCl_2_, 0.2 μM each primer, 0.2 U of AmpliTaq Gold DNA Polymerase (Life Technologies, Carlsbad, CA, USA), and approximately 30 ng of DNA. The amplification products were purified using the QIAquick PCR Purification Kit (Qiagen, Hilden, Germany) and sequenced with the Big Dye Terminator v3.1 Cycle Sequencing Kit using the ABI 3130XL Genetic Analyzer (Applied Biosystems, Waltham, MA, USA). To resolve the ambiguously classified samples, a hierarchical system based on a PCR restriction fragment length polymorphism (PCR-RFLP) protocol using 18 coding region positions was used for haplogroup assignment, as described elsewhere [18].

The sequences were edited using ABI PRISM SeqScape v.2.5. The sequences of the HVS I and HVS II regions for each individual were aligned manually using MEGA 6 and compared with the revised Cambridge Reference Sequence rCRS [19, 20].

We amplified the two HVSI and HVSII segments in different PCRs (thermal cyclers) and corroborated the haplogroup assignation of two researchers to reduce the probability of the artificial recombination. The sequences were corroborated based on the forward and reverse sequencing results.

Haplogroup assignments were based on the HVS I and HVS II sequences of all samples in this study and were determined using online programs, i.e., HaploGrep [21], Mitomap [22], and Phylotree (mtDNA tree Build 16) [23].

### Accession numbers

The control region sequences of the 714 subjects in this study are available at GenBank under the following accession numbers: KT945272–KT945497 (Wallachia group), KT945940–KT945993 (Dobrudja group), KT945705–KT945939 (Moldavia group), and KT945498–KT945704 (Transylvania group).

#### Statistical analysis

Gene diversity parameters, including the number of sequences, haplotype diversity, nucleotide diversity, number of polymorphic sites, and mean number of pairwise differences, were estimated using DnaSP version 5. To assess neutrality, Tajima’s *D* [24] and Fu’s [25] were calculated using ARLEQUIN version 3.5.2.2 [26].

The distance matrix generated by pairwise *F*_ST_ values between populations was calculated with ARLEQUIN version 3.5.2.2 (based on mtDNA haplogroup frequencies) and visualized by a multidimensional scaling (MDS) plot using SPSS 17.0 (SPSS Inc., Chicago, IL, USA). Statistical significance of *F*_ST_ values was assessed using 10000 permutations.

Romanian populations were compared with published data for two groups corresponding to European and Middle East populations (data included in Additional file 2: Table S3).

A principal component analysis (PCA) was performed based on mtDNA haplogroup frequencies using the same software for comparisons among populations. A median joining network (MJN) for certain haplogroups was generated to infer phylogenetic relationships among the populations from the four Romanian provinces using Network v4.5.0.0 (available at http://www.fluxus-engineering.com). The MJN was obtained to better differentiate the haplotype frequencies among the Romanian provinces. Different mutation weights were applied in accordance with the previous papers [27–29], and point insertions and deletions were excluded from the analysis.

Lastly, the Surfer 9.0 application (Golden Software Inc., Golden, CO, USA) was used to graphically represent the geographical distribution of haplogroup frequencies (H, U, HV, T, J, K, X, N, M, V, and W) in the European region by applying the Kriging algorithm for the surface interpolation map.

## Results

We analyzed 714 samples to estimate genetic diversity in each of the four Romanian provinces (Additional file 1: Table S1). We observed high mtDNA haplotype diversity in the Wallachian population. Analyzing the control region, we detected 189 haplotypes and k = 4.530 nucleotide differences, on average. The results are summarized in Additional file 3: Table S2.

We observed similar haplotype and nucleotide diversity values for all Romanian provinces, with a slightly lower nucleotide diversity in the Dobrudja population and higher haplotype diversity in Transylvania. We observed significant deviations from neutrality tests according to Tajima’s D and Fu’s [30] for all Romanian provinces (Additional file 3: Table S2).

We observed length heteroplasmy in HVSI generated by a T to C transition in the poly-C tract between nucleotide positions 16184 and 16193 in 82 individuals (11.48%). In addition to length heteroplasmy, we found two sequence heteroplasmies in HVSI at position 16084 T/C in one sample and at position 16237 T/C in a second sample.

The resampling analysis for the Dobrudja sample using a 1000 replicate bootstrap indicated that the mean statistics for our data in the vector that we analyzed was 3.833. The bootstrap bias was only -0,0152 and its standard error was about 1.1364. The 95% confidence for mean statistic: the lower bound was 2.08 and the upper bound was 6. The histogram of men of bootstrap samples was nearly normal considering the great number of bootstrap replications, as depicted in the Additional file 4: Figure S4.

As expected, we were able to classify the huge majority of individuals from the four Romanian populations into nine Eurasian mitochondrial haplogroups (H, U, K, T, J, HV, V, W, and X). All mtDNA data are summarized in Additional file 1: Table S1.

The Romanian populations also exhibited sequences that belonged to the most frequent Asian haplogroups (haplogroups A, C, D, I, M, and N) and African haplogroup L. We detected haplogroups A, C, D, and I in the Romanian sample, with an overall frequency of 2.24%, consistent with the frequency in other European populations. We observed a relatively high frequency of Asian haplogroups M and N in Wallachia, Dobrudja, and Moldavia, but not in Transylvania, which also lacked the M haplogroup. The haplogroup X, entirely represented by subhaplogroup X2, was present at the highest frequency in Transylvania.

**Fig. 1:**
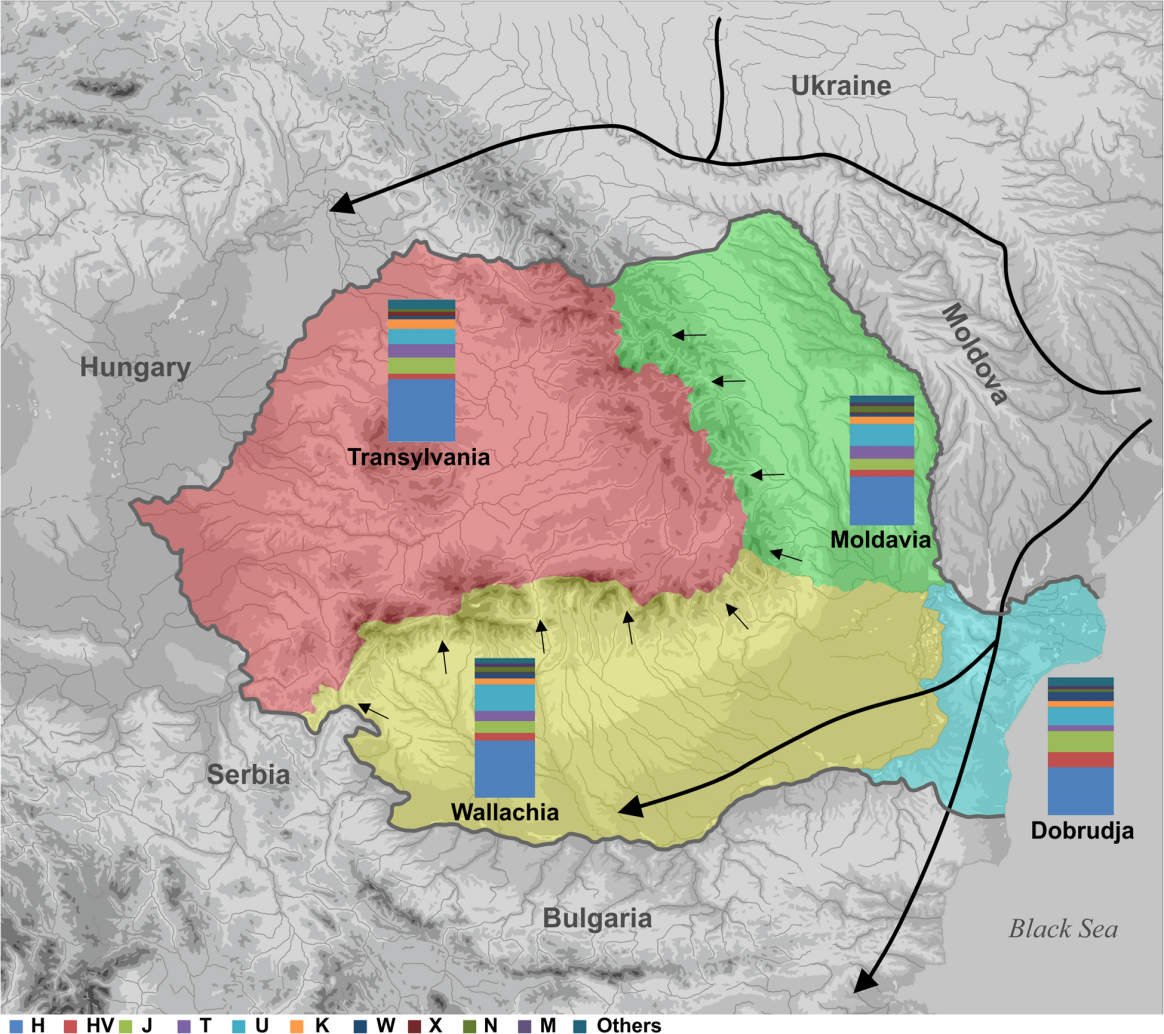
Map of Romania showing the approximate migration routes and the mtDNA haplogroup distribution in the Romanian provinces. The map depicts the geographic distribution of mtDNA haplogroups in the Romanian provinces as reported in Additional file 2: Table S3: Wallachia (*yellow*), Dobrudja (*blue*), Moldavia (*green*) and Transylvania (*red*). Arrows indicate the approximate migration routes running east to west direction that seemed to be used throughout the history since Neolithic period to Middle Ages. The map was constructed by M.C. and R.C

The overall frequency of haplogroup H (40.98%), the most common haplogroup in Europe [31], was consistent with the frequency observed in most European populations, and varied from a relatively high frequency in Transylvania to a lower frequency in Dobrudja, as presented in Fig. 1. Two subhaplogroups, H1 and H2, were quite frequent (>5%), while H3 had a frequency of 0.54%, comparable to previous estimates [13, 32].

Our data indicated that haplogroup U is the second most frequent in all analyzed populations but is noticeably less frequent in Transylvania than in other areas (Fig. 1). In our sample, we detected the subhaplogroups U1, U6, and U7 at a general frequency of 1.4%, 0.14% and 0.14%, respectively. Within haplogroup U, the most ancient and prevalent subhaplogroup in Europe [33], U5 had the highest frequency (47.94% within haplogroup U) in all four provinces.

In Walachia, Moldavia, and Transylvania, we detected similar HV haplogroup frequencies to those observed in other European countries, but we observed the highest frequency in the Dobrudja population. With respect to haplogroups N, M, X, and V, we found different frequency distributions between Transylvania and the other three provinces, as depicted in Additional file 2: Table S3 and Fig. 1.

**Fig. 2:**
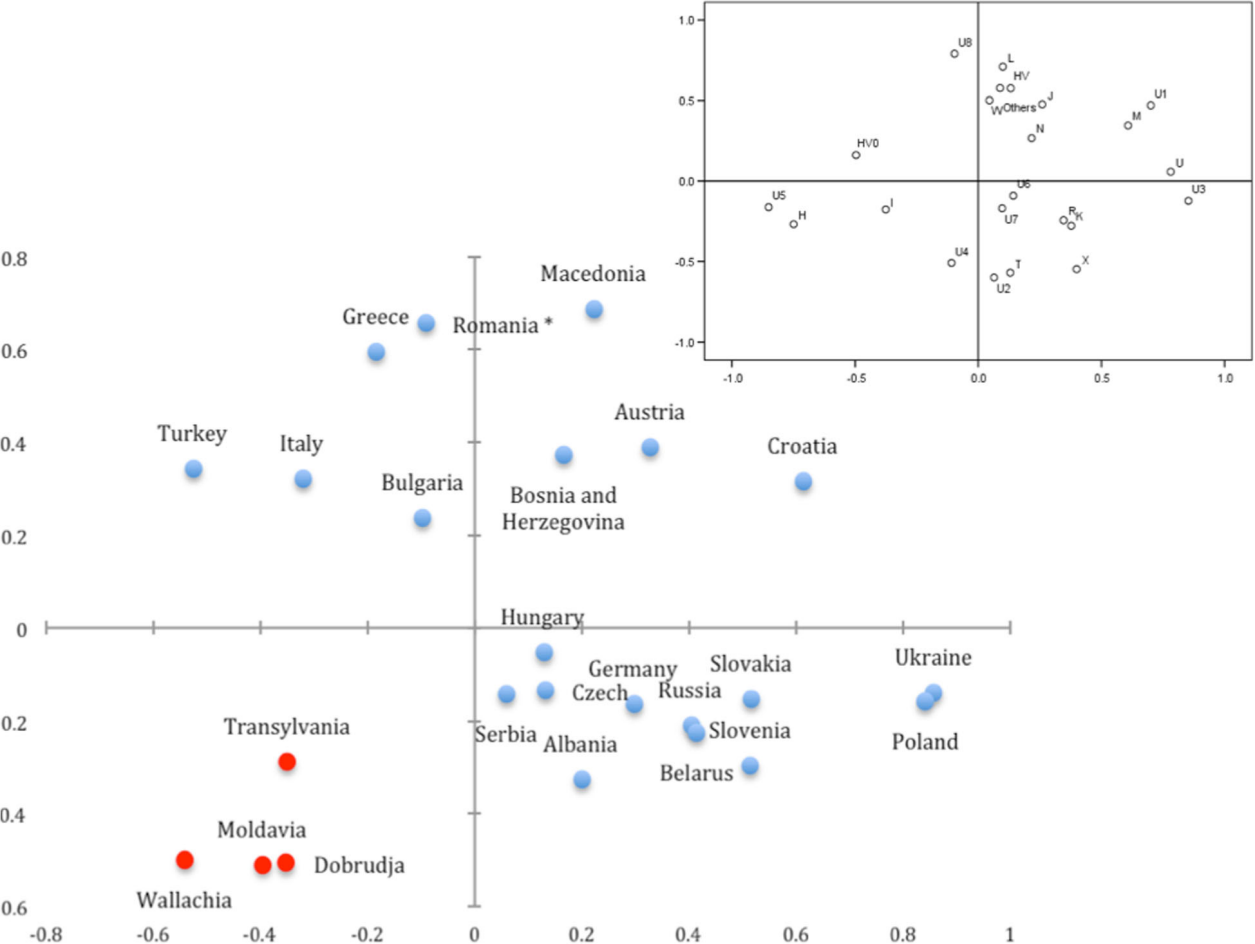
Principal Component Analysis of mitochondrial haplogroup frequencies of the neighboring and Romanian provinces populations. The Romanian provinces in the present study are represented in *red* dots. The Romanian population marked with the asterisk sign refers to previously published data as in the Additional file 2: Table S3. The upper right plot shows the correlation of each haplogroup to the first and second axes of PCA

To explore the genetic affinities of Romanian populations with neighboring populations, we conducted a PCA based on the frequencies of mitochondrial haplogroups (Fig. 2). The first component (PC) accounted for 17.34% of the total haplogroup variation (30.68%) and separated the European populations into roughly three clusters, with the Romanian provinces forming an individual group with Transylvania close to the center of the axis. The second PC accounted for 13.38% of the total haplogroup variation and did not clearly distinguish populations, although the Romanian provinces formed a single cluster, with Wallachia slightly dissociated from these provinces at the end of this vector. Component loadings of PC1 indicated high correlation coefficients for M, U1, and U5 (0.689, 0.840, and -0.759, respectively), supporting the grouping of the analyzed populations (Fig. 2).

The high frequencies of the haplogroup M (excepting Transylvania) and U5, as well as the lower frequencies of subhaplogroup U1 reveal a pattern that could explain the distribution of Romanian populations on the PCA plot. Component loadings of PC2 indicated a high correlation coefficient for X, which is consistent with the observed high frequencies of the haplogroup X in the Wallachian, Dobrudjan, and Moldavian provinces.

To further visualize the relationships among Romanian populations analyzed here and European and Near Eastern populations, we estimated pairwise *F*_ST_ based on the mitochondrial haplogroup frequencies for 19 neighboring populations, including Romanian mtDNA data from previous studies, or 41 populations from all of Europe and the Near East (data shown in Additional file 2: Table S3). For both analyses, the pairwise population *F*_ST_ values were not statistically significant (*p* = 0.00000 ± 0.0000, *F*_ST_ test), indicating no differentiation among Romanian provinces (Additional file 5: Table S4). In a statistical analysis, we only found statistically significant differentiation (*p* = 0.00000) between these provinces and the Caucasus, Egypt, or Turkey populations.

**Fig. 3:**
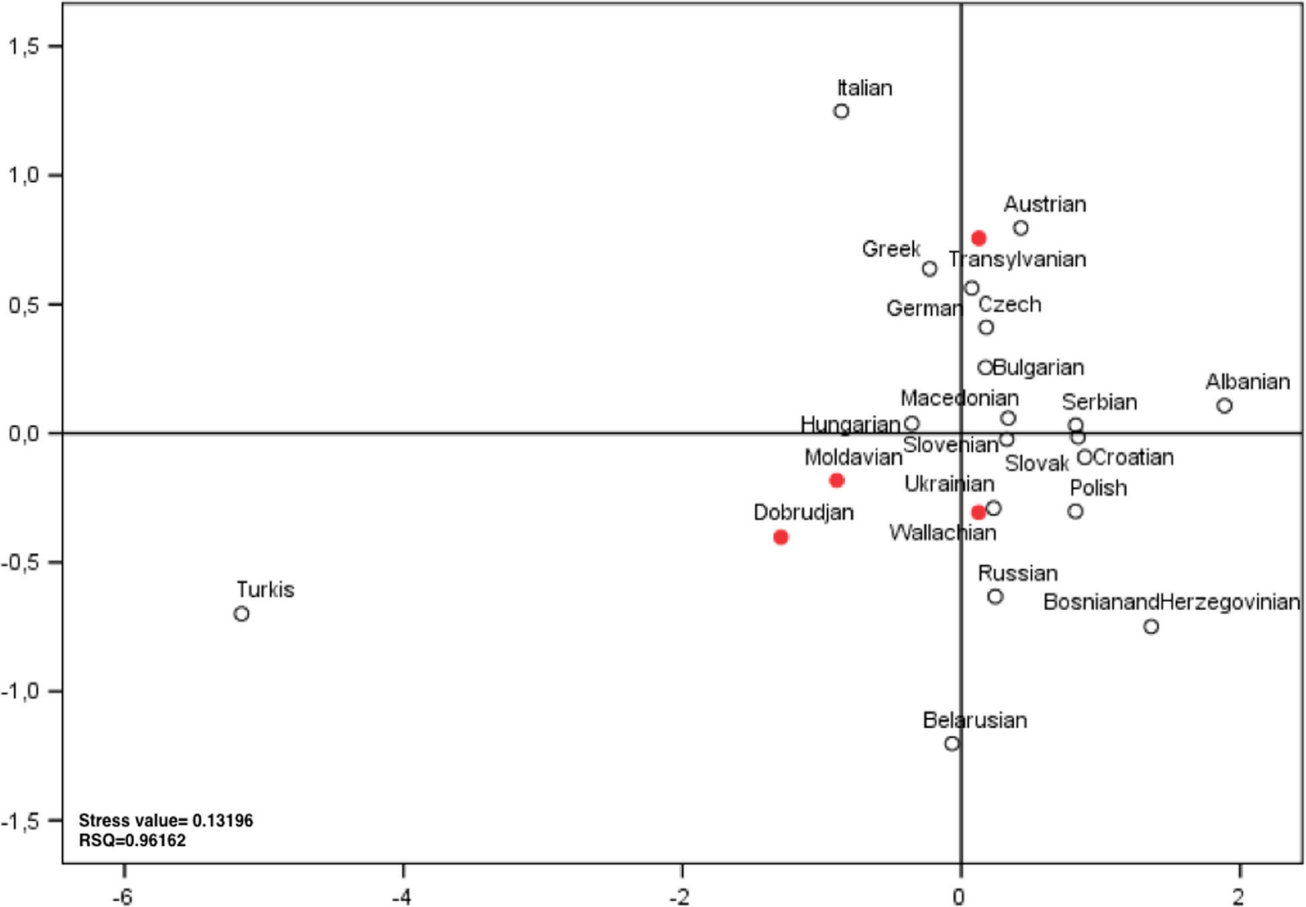
Multi-dimensional scaling plot of pairwise F_ST_-values of the Romanian populations and other 19 neighboring populations. The F_ST_ genetic distances were calculated based on the mtDNA haplogroup frequencies of different populations: Romanian provinces (in *red*) and neighboring populations (*blank circles*)

Based on MDS plots including the geographical neighbors or 41 populations, we found that Transylvania is more closely related to Central European populations than to the other Romanian provinces, which are more closely related to the Balkan populations (Fig. 3 and Additional file 6: Figure S1).

**Fig. 4:**
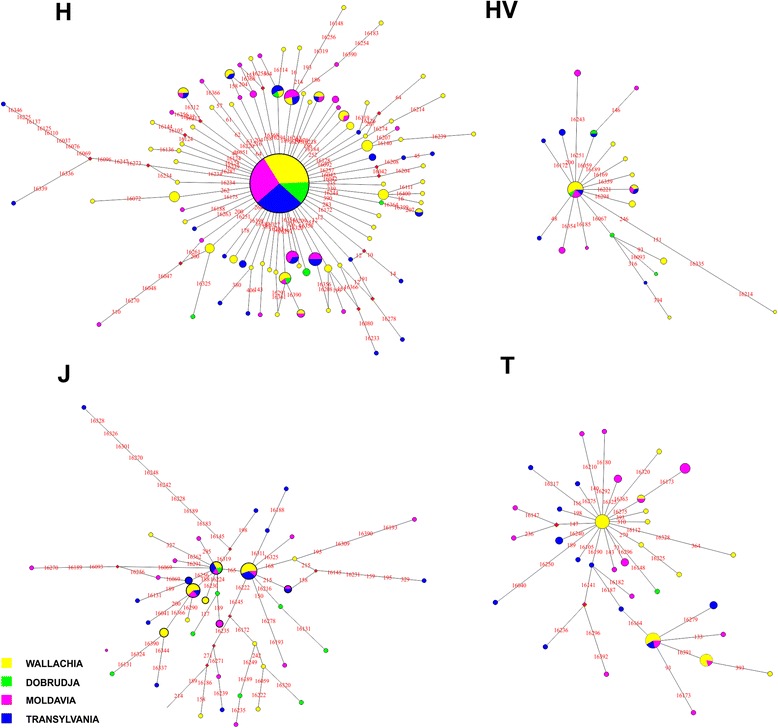
Specific median-joining network of haplogroups H, HV, J and T in the Romanian provinces. Data encompass mtDNA HVS I (positions 16024-16416) and HVS II (positions 1-410)

To establish the relationships among populations based on control region sequences, we built a MJN based on HVSI and HVSII sequences for all major haplogroups (H, U, HV, T, J, K, X, I, N, M, V, and W; Fig. 4 and Additional file 7: Figure S2). The majority of haplotypes shaping the networks formed a star-like phylogeny, while others, such as haplogroups U, N, and I, did not. The network topology did not reveal a major cluster of Romanian sequences within the all networks.

As anticipated, a substantial number of haplotypes observed in all Romanian provinces generated a main haplogroup H cluster with a star-like shape. In general, we detected a large number of haplotypes that were shared among Wallachia, Moldavia, and Transylvania populations. In particular, we detected haplotypes that were shared between Moldavia and Wallachia as well as between Moldavia and Transylvania. We observed high sequence diversity within haplogroup U and no star-like topology; the haplotypes were mostly shared between Moldavia and Transylvania and between Moldavia and Wallachia according to the majority of the networks. For Dobrudja, we observed shared haplotypes with Moldavia and Wallachia.

**Fig. 5:**
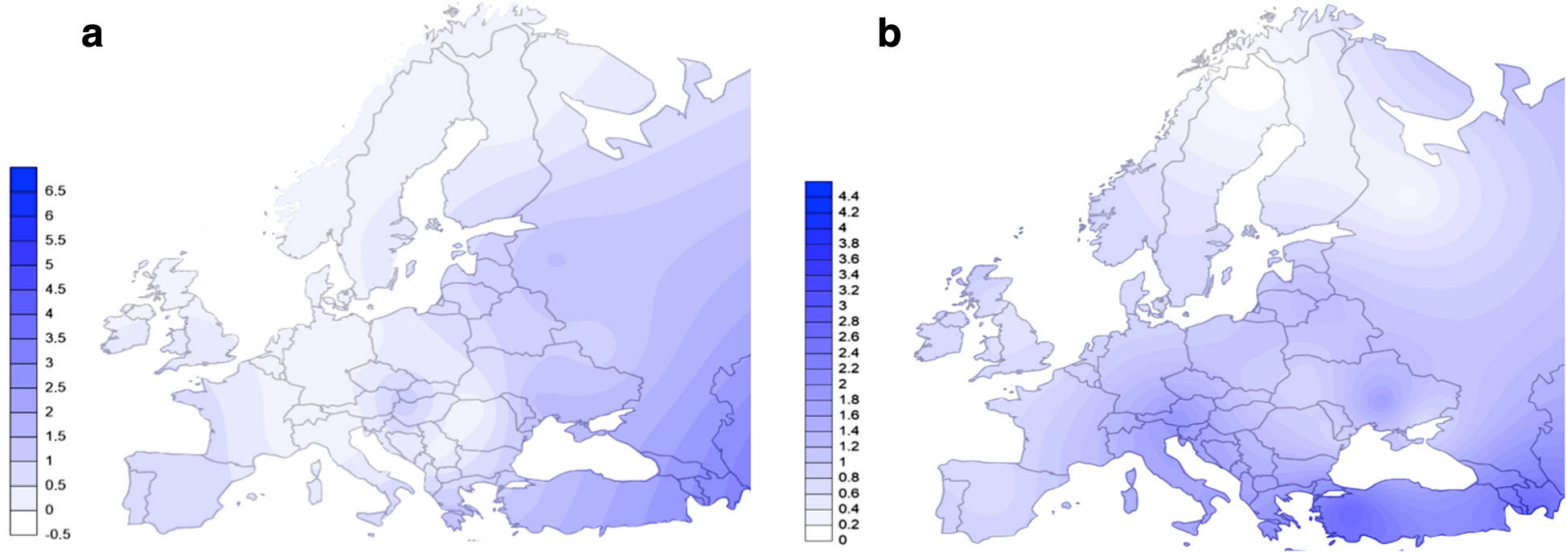
Interpolation frequency maps of lineages M and X across Europe and Near East. The interpolation maps were created using the Kriging algorithm showing the spatial frequency distribution of: (**a**) haplogroup M and (**b**) haplogroup X, as obtained in our data set and literature listed in the Additional file 2: Table S3. Darker blue shading corresponds to the higher frequencies of the shown haplogroups. The figure was constructed by R.C. and F.R. using the Surfer 9.0 application (Golden Software Inc., Golden, CO, USA)

The overall spatial distribution of haplogroup frequencies indicated higher frequencies of the H, V, and X haplogroups and lower frequencies of U and N in Transylvania than in the other three provinces (Fig. 5 and Additional file 8: Figure S3). We did not detect haplogroup M in Transylvania.

## Discussion

Our analysis of mitochondrial DNA control region variation for 714 samples confirmed the genetic affinity of all historical provinces to Southeastern European populations and demonstrated that Transylvania samples were most closely related to those from central Europe.

We observed similar frequency distributions for the majority of mitochondrial haplogroups among the four Romanian provinces. For other haplogroups, we detected a variation in frequencies, with overall values within the range for central and Southeastern European populations.

As highlighted by their spatial frequency distribution, we detected differences in mtDNA haplogroup frequencies among the populations of these four Romanian provinces. We observed general genetic differentiation of the Transylvania population as a whole, considering some haplogroups had higher or lower frequencies in this region than in the other three provinces (Fig. 5 and Additional file 8: Figure S3). Geographical position and proximity to Central European populations, together with historical gene flow determined by the topology of Romania could explain these data.

The genetic affinities, illustrated by the mtDNA haplogroup frequencies, among the four Romanian provinces were supported by a PCA plot in which these populations formed a clustered, and Transylvania was slightly detached from this cluster. The location of this cluster in the PCA analysis, i.e., separated from the other European populations, is in line with the results of an important study based on SNP markers showing that Romanians cluster with other Eastern European populations and providing evidence for complex admixture from Southwestern Asia [34].

The location of Transylvania in the European space was more evident in both MDS analyses, which are based on pairwise *F*_ST_, indicating a noticeable genetic proximity of this province to Central European populations, in contrast to the other three provinces, which show spatial integration within the Balkan populations.

These observations are consistent with previous analyses of the non-recombining region of the Y chromosome (NRY), suggesting that the Carpathians correspond with a genetic boundary, as evidenced by a discontinuity of NRY markers between the East and West of the Carpathian ridge, highlighted by the peculiarities of samples from Central Transylvania compared to Moldavia [35, 36].

The networks that show the general topology of control sequences of Romanian samples mainly indicate overlapping haplotypes between Moldavia-Wallachia and Moldavia-Transylvania, suggesting gene flow between these provinces. The substantial workforce movement from Moldavia towards these two provinces throughout the communist period supports these observations. The geographic proximity and recent population movement explain the common haplotypes observed in Dobrudja, Moldavia, and Wallachia.

The differences in the haplogroup frequency distributions and haplotype diversity in Dobrudja can also be explained by its smaller sample size compared to that of the other populations included in the study.

The presence of Asian mtDNA lineages among Romanian populations, albeit at low frequencies, indicates the flow of maternal lineages along migration routes through East Asia and Europe during different time periods, namely, the Upper Paleolithic period and/or, with a likely greater preponderance, the Middle Ages [37, 38]. It is worth mentioning that a higher frequency value of these lineages (7.9%) was earlier reported in two Hungarian ethnic groups in Transylvania, and this was attributed to their genetic proximity to west Eurasian populations [14]. This clear discrepancy between Romanian populations and Hungarian minority groups could reflect a reduction in admixture, as was historically documented later for the Saxons in Romania [7]. This contribution of genes from Southwestern Asia is not supported by our pairwise *F*_ST_ values, which indicate statistically significant differentiation between Wallachian, Moldavian, and Transylvanian populations and the Asian population included in the analysis. The Dobrudjan population did not show the same patterns; a larger Asian influence shaped genetic variation in this province during its history*.*

As for haplogroup L, we found two samples with L3, one in Wallachia and the other in Dobrudja (0.28%), consistent with the low frequencies found in European populations [39], except in the Iberian Peninsula, where Western Andalusians have high frequencies of African lineages [40]. The occurrence of the African haplogroup L might be the result of colonists from African provinces during the Roman period or the recent slave trade that generated movements of African people from the Ottoman Empire to East Europe at the beginning of the 17^th^ century.

The different genetic affinities observed here could be explained by the topology of the Romanian territory, which influenced admixture among the Transylvania population and neighboring populations over time, together with the distinct historically attested migration [11] and colonization events [12], which were likely the main determinants of the close genetic resemblance of Transylvania and Central European populations. In this scenario, the Carpathian Arch could have played a defining role for the major three migration routes that were apparently used throughout history, since the Neolithic period to the Middle Ages. One route was to the Baltic region and the Northern Pontic steppe and, from there, through the Trans-Carpathian passes in the present-day Ukrainian Carpathians, towards the Tisza plain and Transylvania. The second route crossed Wallachia, Dobruja, and Moldavia, and was related to the lower Danube River basin and Black Sea, which connected the basins of the Prut, Dniester, and lower Danube to northern Europe, the Northern Pontic steppes, and the Balkan Peninsula. The third was through the passes of the Eastern and Southern Carpathians (Fig. 1). The first two routes were highly active during prehistory, the Roman period, Migration period, and Middle Ages, as evidenced by archaeological findings, and the Carpathians passes were rarely exploited for large-scale population movements.

## Conclusions

In our mtDNA analysis, we observed a uniform distribution of the majority of haplogroups among Romanian provinces with the populations in the Wallachia, Moldavia, and Dobrudja provinces tended to cluster together within the Slav population based on the MDS scatterplots, while Transylvania was more closely related to Central European populations. We confirmed gene flow from Southwestern Asia based on pairwise population *F*_ST_ values strictly in the case of Dobrudja, and this result could be easily explained by the greater Asian influence in this area throughout history.

Our findings suggesting a closer affinity of Transylvania to central Europe are supported by the observed haplogroup frequency clines for several European and Asian maternal lineages and could be attributed to different migratory routes shaped by the Carpathian Arch.

The mitochondrial DNA data in this study and the analysis of differentiation among historical regions represent a considerable extension of our knowledge of mitochondrial diversity in Romania.

## Additional files

Additional file 1: Table S1. Contains the detailed description of mitochondrial molecular data of the 714 Romanian sample in the present study. (XLSX 103 kb)
Additional file 2: Table S3. List of absolute frequencies of mtDNA haplogroups and sub-haplogroups in the 24 populations and 45 populations used for the PCA and MDS analyses. (XLSX 63 kb)
Additional file 3: Table S2. Diversity indices and neutrality test for the Romanian historical provinces based on the HVSI and HVS II sequences. (XLSX 34 kb)
Additional file 4: Figure S4. The resampling analysis for the Dobrudja sample using a 1000 replicate bootstrap. (DOCX 32 kb)
Additional file 5: Table S4. The Fst analyses based on the populations of Europe and Near East analyses. (XLSX 64 kb)
Additional file 6: Figure S1. Multi-dimensional scaling plot of pairwise F_ST_-values of Romanian populations and 41 populations of Europe and Near East. (DOCX 59 kb)
Additional file 7: Figure S2. Specific median networks of haplogroups U, K, M, N, W, V, X, and I. (PDF 1820 kb)
Additional file 8: Figure S3. Interpolation frequency map of haplogroups H, HV, U, K, T, J, N, and W. The figure was constructed by R.C. and F.R. using the Surfer 9.0 application (Golden Software Inc., Golden, CO, USA). (PDF 24007 kb)

## Abbreviations

AD: After death
BP: Before Christ
Cca: Circa
DNA: Deoxyribonucleic Acid
HVS: Hypervariable regions
Kya: Thousand years ago
LGM: Last glacial maximum
LKB: Linearbandkeramik
MDS: Multidimensional scaling
MJN: Median joining network
mtDNA: Mitochondrial DNA
NRY: Non-recombining part of the Y chromosome
PCA: Principal component analysis
PCR-RFLP: Polymerase chain reaction-restriction fragment length polymorphism
rCRS: Cambridge reference sequence
SNP: Single nucleotide polymorphism
