# Supplementary material for: Genetic affinities among the historical provinces of Romania and Central Europe as revealed by an mtDNA analysis
Source: BMC Genet. 2017 Mar 7;18:20. doi: 10.1186/s12863-017-0487-5 (PMC5341396; doi:10.1186/s12863-017-0487-5)

**Additional file 4: Figure S4**. The resampling analysis for the Dobrudja sample using a 1000 replicate bootstrap. The probability was constructed using a ten set of bins based on the bootstrap mean frequency. The histogram of men of bootstrap samples was nearly normal considering the great number of bootstrap replications.


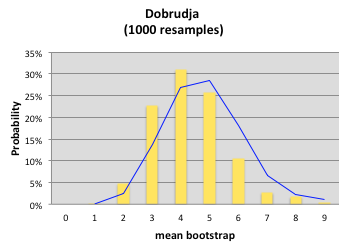

Supplement: Additional file 4: Figure S4. — The resampling analysis for the Dobrudja sample using a 1000 replicate bootstrap. (DOCX 32 kb) [file 12863_2017_487_MOESM4_ESM.docx]
