# Supplementary material for: Genetic affinities among the historical provinces of Romania and Central Europe as revealed by an mtDNA analysis
Source: BMC Genet. 2017 Mar 7;18:20. doi: 10.1186/s12863-017-0487-5 (PMC5341396; doi:10.1186/s12863-017-0487-5)
Supplement: Additional file 6: Figure S1. — Multi-dimensional scaling plot of pairwise FST-values of Romanian populations and 41 populations of Europe and Near East. (DOCX 59 kb) [file 12863_2017_487_MOESM6_ESM.docx]

**Additional file 6:Figure S1**. Multi-dimensional scaling plot of pairwise FST-values of the Romanian populations and 41 populations of Europe and the Near East. The Romanian provinces (in red) and the 41 populations (blank circles).


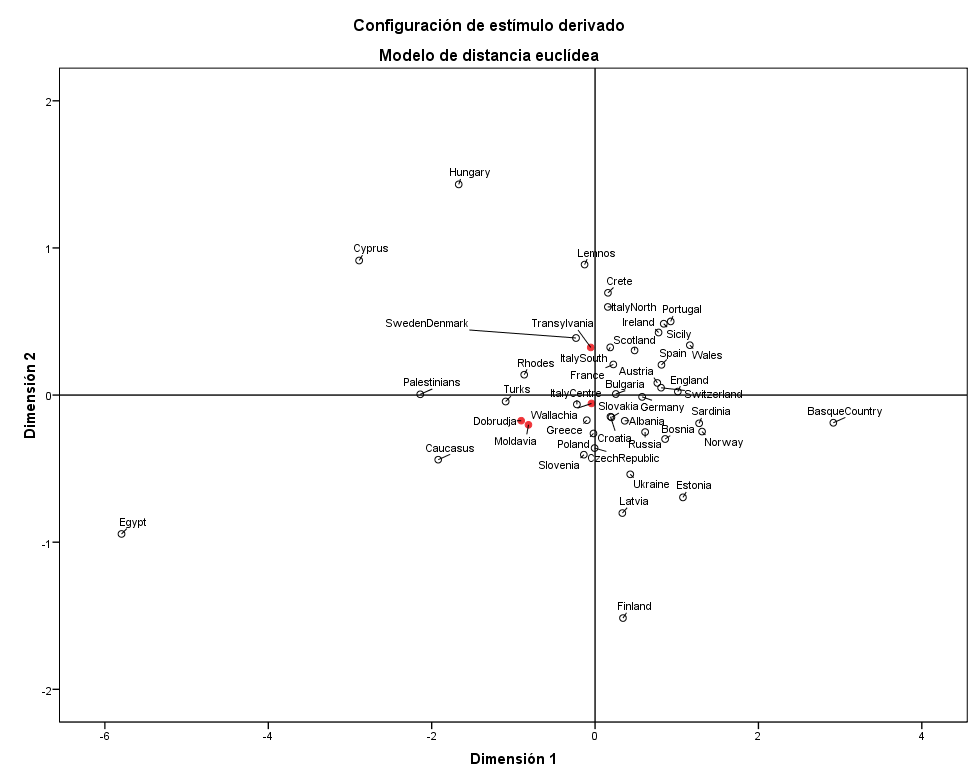


RSQ =0.96788

Stress value=0.11915
