# Supplementary material for: Genetic affinities among the historical provinces of Romania and Central Europe as revealed by an mtDNA analysis
Source: BMC Genet. 2017 Mar 7;18:20. doi: 10.1186/s12863-017-0487-5 (PMC5341396; doi:10.1186/s12863-017-0487-5)
Supplement: Additional file 7: Figure S2. — Specific median networks of haplogroups U, K, M, N, W, V, X, and I. (PDF 1820 kb) [file 12863_2017_487_MOESM7_ESM.pdf]

Additional file 7: Figure S2 Median Joining Networks of haplogroups U, K, M, N, W, V, X and I. Data encompass mtDNA (nps 16024-16416) and HVS II (nps 1-410).

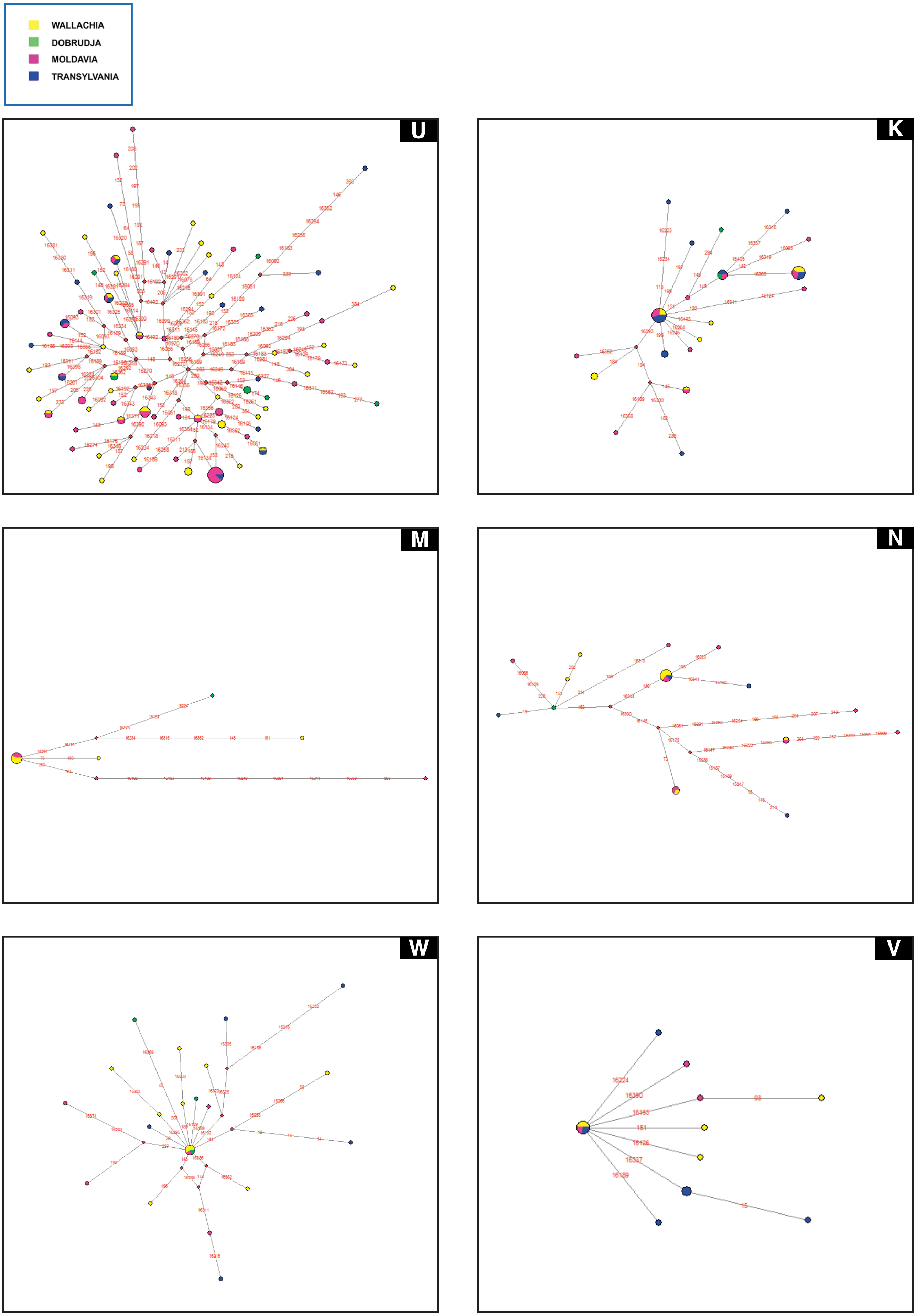

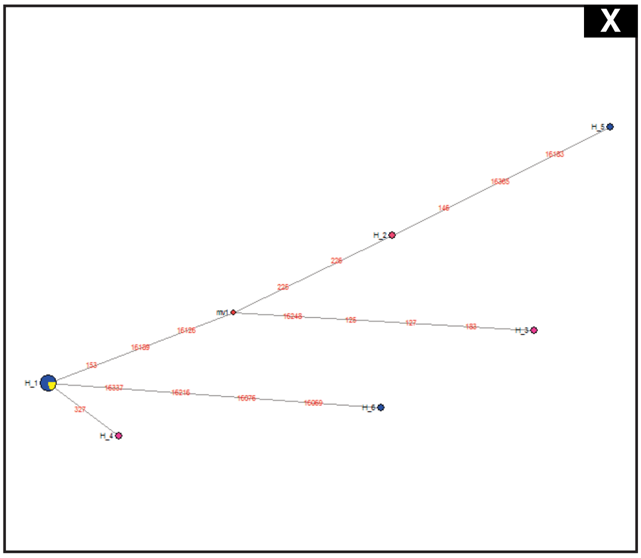
X
H_1
H_2
H_3
H_4
H_5
H_6
mv1
153
16189
16126
225
226
146
16365
16183
16248
126
127
183
16337
16216
16076
16069
327

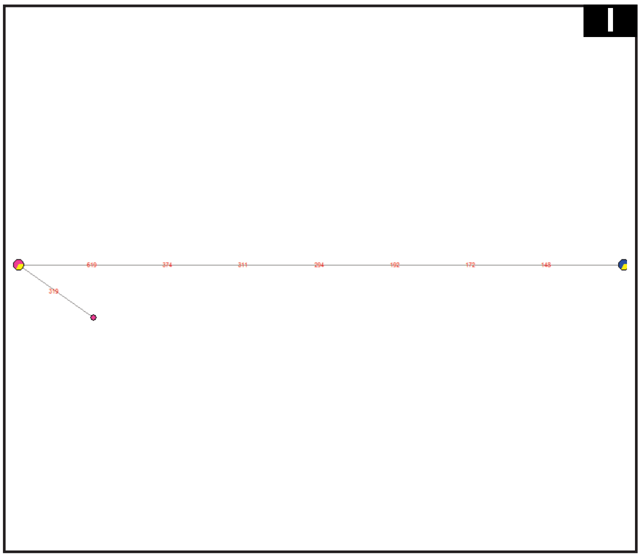
I
619
374
311
294
192
172
148
319
