# Supplementary figures and images for: Genetic affinities among the historical provinces of Romania and Central Europe as revealed by an mtDNA analysis
Source: BMC Genet. 2017 Mar 7;18:20. doi: 10.1186/s12863-017-0487-5 (PMC5341396; doi:10.1186/s12863-017-0487-5)

**Additional file 8:** Figures S3 Interpolation frequency map of haplogroups H, HV, U, K, T, J, N and W.

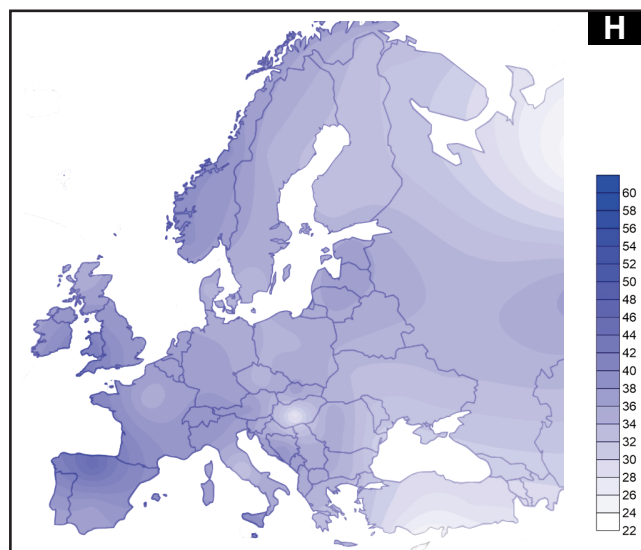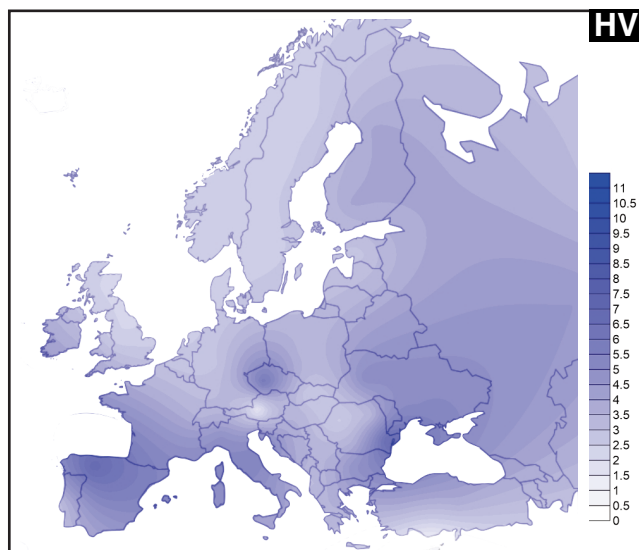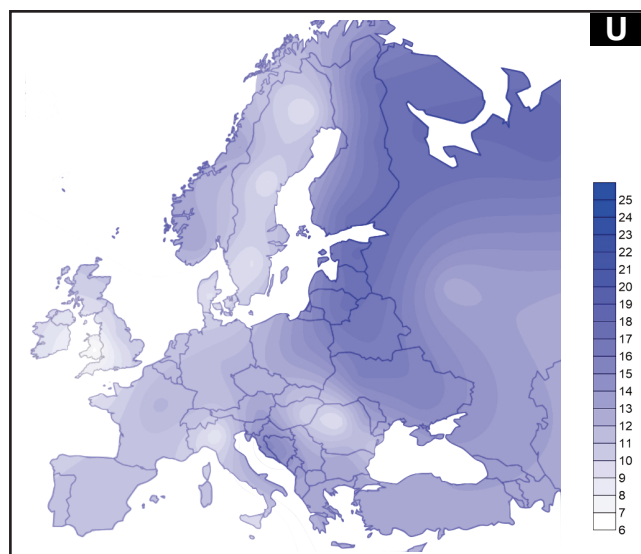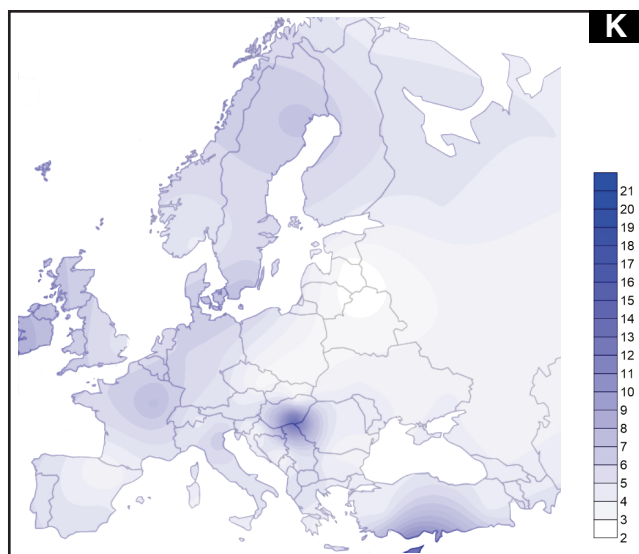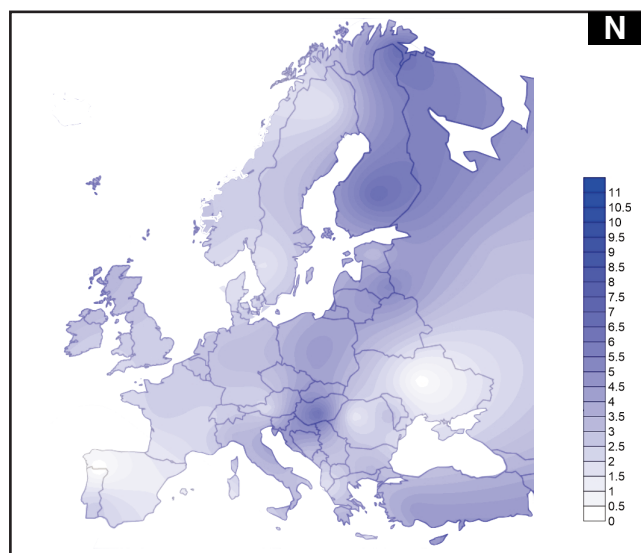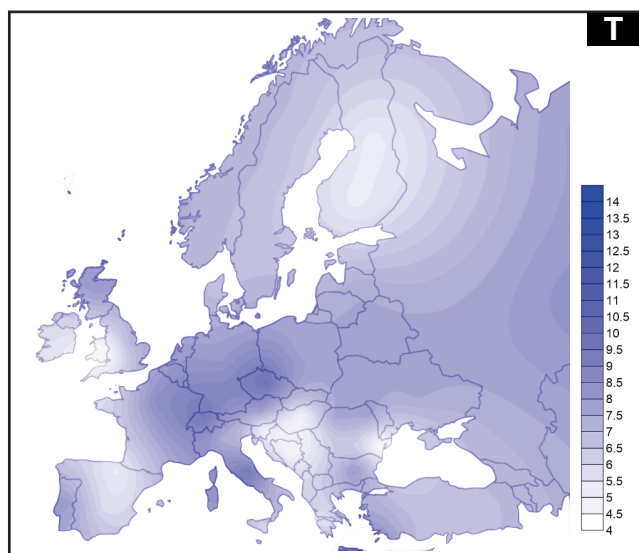

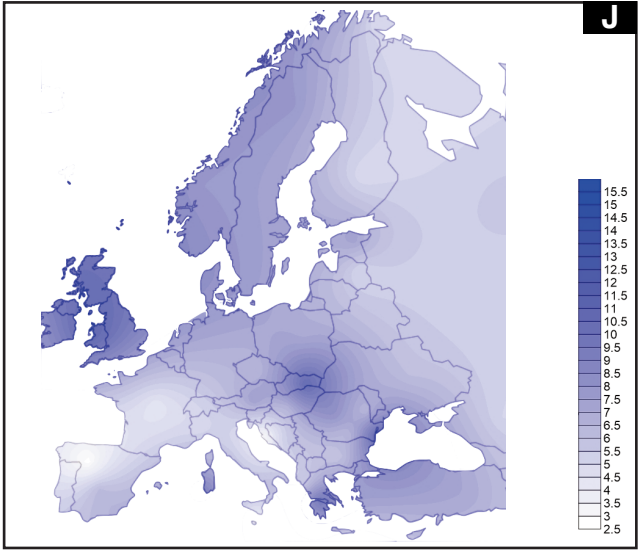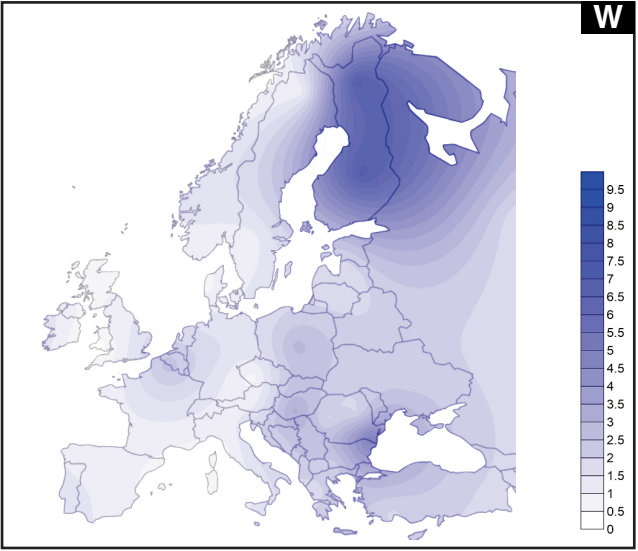

Supplement: Additional file 8: Figure S3. — Interpolation frequency map of haplogroups H, HV, U, K, T, J, N, and W. The figure was constructed by R.C. and F.R. using the Surfer 9.0 application (Golden Software Inc., Golden, CO, USA). (PDF 24007 kb) [file 12863_2017_487_MOESM8_ESM.pdf]
